# Listerine

**Published:** 1883-09

**Authors:** 


### LISTERINE.

Although this is not a new remedy, yet we believe its use in dentistry is comparatively novel. It is largely employed as an antiseptic for lotions and for surgical dressings in medical practice, and it will be found quite as useful in the dentist's case. We have for some time been using it in carious teeth, for dressing operative wounds in the mouth, and as a gargle in cases of ulcerative sore throat. We have injected it into alveolar abscesses, and have employed it to neutralize an offensive breath in patients. It is especially grateful as a mouth-wash after the extraction of teeth. It may be employed in full strength, or in various degrees of dilution with water. It is not a secret preparation, but its formula may be found printed upon the label of every bottle. We commend it to the use of dentists.

